# Intraoperative methylene blue testing for female urethral diverticulum: diagnostic value and surgical outcomes

**DOI:** 10.3389/fruro.2025.1735050

**Published:** 2026-01-06

**Authors:** Lateefa Aldakhil

**Affiliations:** Department of Obstetric and Gynecology, King Khalid University Hospital, King Saud University, Riyadh, Saudi Arabia

**Keywords:** diagnostic delay, female urology, methylene blue, recurrent urinary tract infection, urethral diverticulectomy, urethral diverticulum, urogynecology

## Abstract

**Background:**

Female urethral diverticulum (UD) is a rare and often underdiagnosed condition that mimics other lower urinary tract disorders, leading to diagnostic delays. This study evaluated the clinical presentation, diagnostic accuracy, and surgical outcomes of methylene blue-assisted cystourethroscopy as an adjunct tool in confirming and localizing UD.

**Methods:**

Fifteen female patients suspected of UD between 2015 and 2025 were retrospectively reviewed. All underwent cystourethroscopy with intraoperative methylene blue dye injection. Diagnostic findings were correlated with final histopathology, and surgical outcomes were assessed following transvaginal diverticulectomy.

**Results:**

Twelve patients (80%) had histologically confirmed UD, while three had non-diverticular lesions (two Skene’s gland cysts and one vaginal mucosa cyst). The methylene blue test was positive in 11 of 12 UD cases, yielding 91.7% sensitivity, 100% specificity, and 93.3% overall diagnostic accuracy. Most diverticula were mid-urethral (66.6%). Postoperatively, 83.3% achieved complete symptom resolution, while recurrence (16.7%) and fistula (8.3%) were successfully managed. No new stress incontinence or urethral stricture occurred.

**Conclusion:**

Methylene blue-assisted cystourethroscopy is a simple, accurate, and low-cost adjunct that enhances intraoperative diagnosis and localization of female UD. It may be helpful in resource-limited settings. However, its role remains adjunctive, as it cannot replace MRI in defining complex anatomy. The small sample size, retrospective design, and inconsistent imaging represent key limitations. Larger prospective studies are needed to validate these findings.

## Introduction

Female urethral diverticulum (UD) is a rare but clinically significant condition characterized by a saccular outpouching of the urethral wall that communicates with the urethral lumen. The reported prevalence ranges from 0.6% to 6%, although the true incidence is likely underestimated due to its variable presentation and frequent misdiagnosis (1–3). Most cases are acquired, resulting from infection or obstruction of the periurethral glands, which may rupture into the urethral lumen and become epithelialized, forming a diverticular sac (4, 5).

The clinical manifestations of UD are notoriously non-specific. While the classical “three D” triad—dysuria, dyspareunia, and post-void dribbling—is considered pathognomonic, it occurs in fewer than 20% of cases (6, 7). The majority of affected women present with recurrent urinary tract infections (UTIs), pelvic or vaginal pain, dyspareunia, or a palpable anterior vaginal wall mass (8–10). Because these symptoms overlap with common lower urinary tract and pelvic floor disorders, misdiagnosis, such as cystocele, urethral cyst, or Skene’s gland abscess, is frequent, often resulting in diagnostic delays exceeding 1 year (11, 12). A careful physical examination remains a crucial first step; expression of purulent or urinary discharge from a tender anterior vaginal wall cyst is considered strongly suggestive of UD (13). However, the absence of such findings does not exclude the condition, particularly in small, complex, or proximally located diverticula. Historically, voiding cystourethrography (VCUG) and ultrasound have been used to confirm diagnosis, but their reported sensitivity ranges only between 65% and 85%, and results are highly operator-dependent (14, 15).

In the past two decades, magnetic resonance imaging (MRI) has emerged as the gold standard for diagnosis, offering a sensitivity and specificity approaching 95%–100% (16–18). MRI provides detailed anatomical information, including the size, location, and configuration of the diverticulum, as well as its relationship to the urethra—parameters essential for preoperative planning. Comparative studies have consistently shown MRI to be superior to ultrasound and VCUG in detecting small or circumferential diverticula and in identifying recurrent lesions (17–19). Nevertheless, MRI may not always be available or feasible, particularly in resource-limited settings. In such circumstances, cystourethroscopy remains an essential diagnostic tool, allowing direct visualization of the diverticular ostium and assessment of associated urethral pathology (20). However, its diagnostic yield alone may be limited, especially when the diverticular opening is small, multiple, or located proximally (21). To overcome this limitation, our study investigates the use of an intraoperative methylene blue test as an adjunct to cystourethroscopy. This simple, low-cost technique enhances both the sensitivity and specificity of endoscopic assessment by facilitating accurate localization of the diverticular neck and confirming its communication with the urethral lumen. By improving intraoperative visualization, the method offers an accessible and reproducible approach to diagnosis and surgical planning, particularly in environments where MRI is unavailable. Definitive treatment of female urethral diverticulum is surgical, with transvaginal diverticulectomy regarded as the therapeutic gold standard, achieving cure rates of 80%–95% and recurrence rates below 10% when performed with multilayer closure and interposition of vascularized tissue (7, 17, 22). However, accurate pre- and intraoperative localization of the diverticular neck remains the most critical factor determining surgical success and minimizing complications such as recurrence and fistula formation (17, 18) and guides preoperative counseling more accurately. The aim was to evaluate the diagnostic utility of the intraoperative methylene blue (MB) test to be used in conjunction with available diagnostic tools.

## Methods

This retrospective descriptive study was conducted at King Saud University Medical City, Riyadh, Saudi Arabia, between January 2017 and December 2025. The study was approved by the Institutional Review Board (IRB) of King Saud University Medical City. We included all female patients with a preliminary preoperative diagnosis of UD. Diagnosis at presentation was based on clinical examination and/or radiological imaging (such as MRI, ultrasound, or voiding cystourethrography).

**Figure 1:**
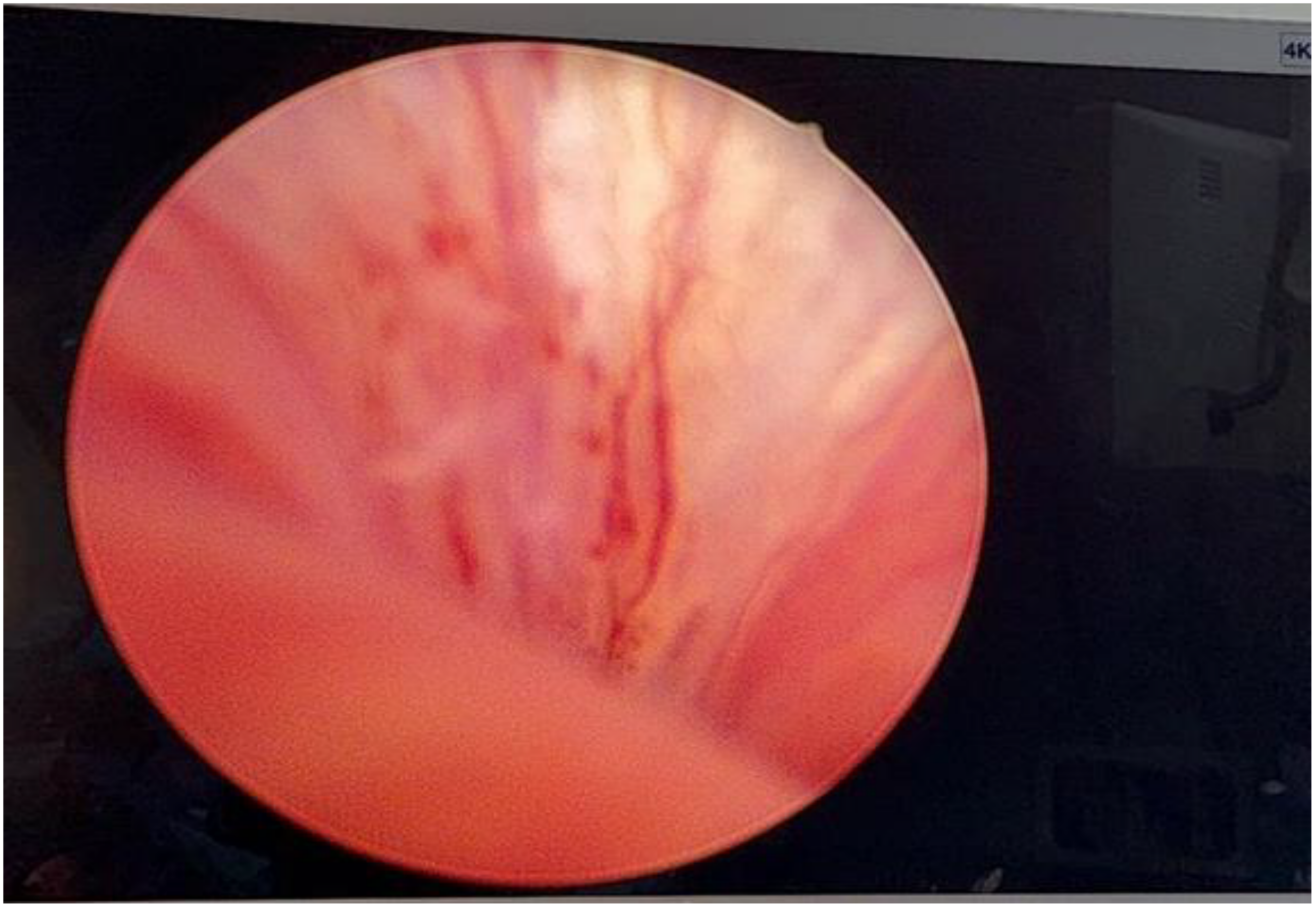
Cystourethroscopy revealed no identifiable urethral diverticulum ostium.

**Figure 2:**
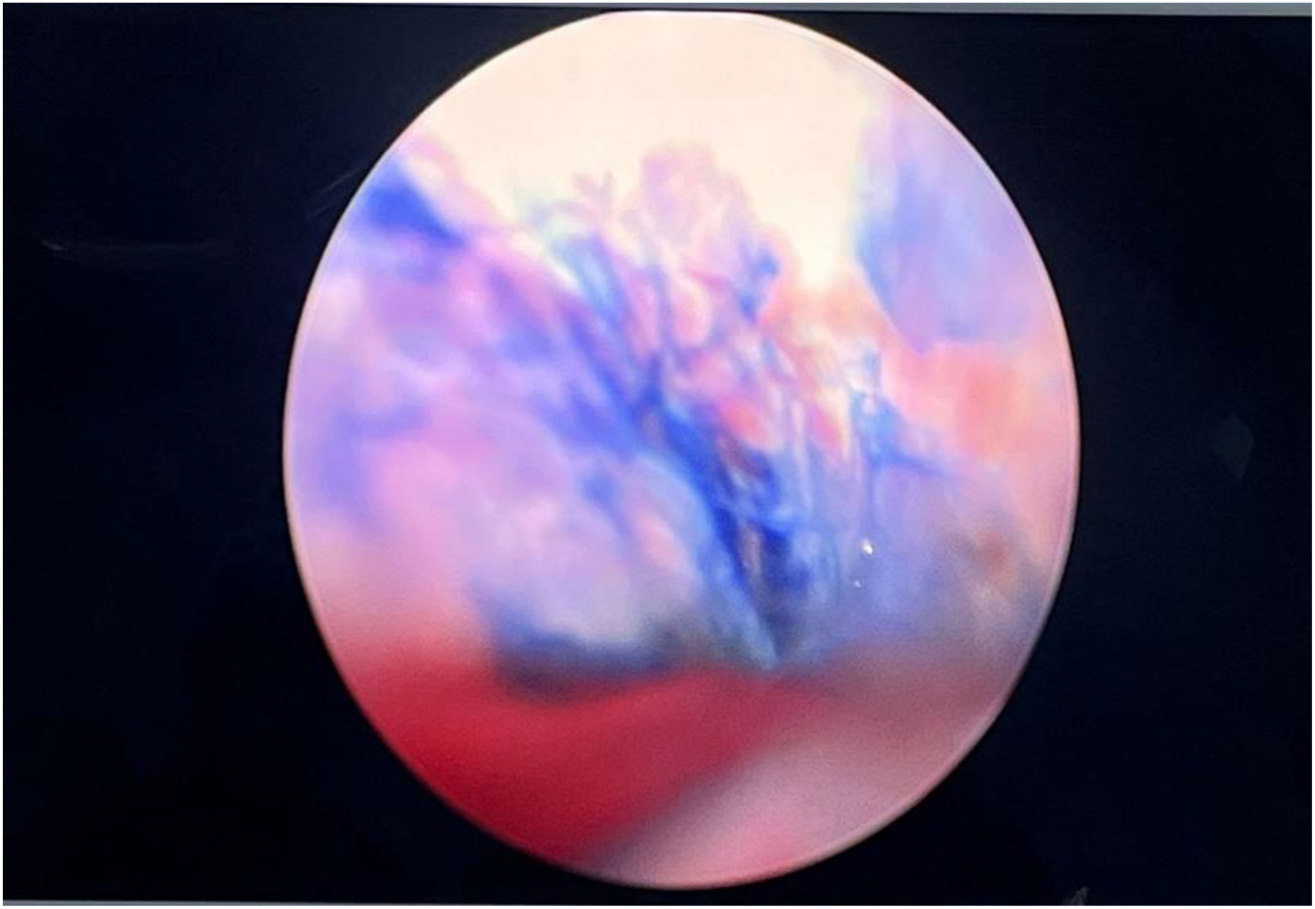
Intraoperative methylene blue (MB) test showing blue-stained fluid passing through the diverticular neck into the urethral lumen, confirming communication.

All included patients underwent diagnostic cystourethroscopy using a standardized methylene blue injection technique to improve visualization and diagnostic yield. The procedure was performed under light sedation or general anesthesia. A 16-French, 0-degree rigid cystoscope was used to evaluate the urethra and bladder. Methylene blue dye was injected into the suspected diverticular cavity using a fine-gauge (insulin) needle transvaginal while performing cystourethroscopy. Gentle manual pressure was applied over the anterior vaginal wall mass, and the appearance of blue-stained fluid in the urethral lumen confirmed the diagnosis and communication (**Figures 1**, **2**). The location of the diverticular opening was documented as proximal, middle, or distal according to its relationship to the urethral length and bladder neck.

**Figure 3:**
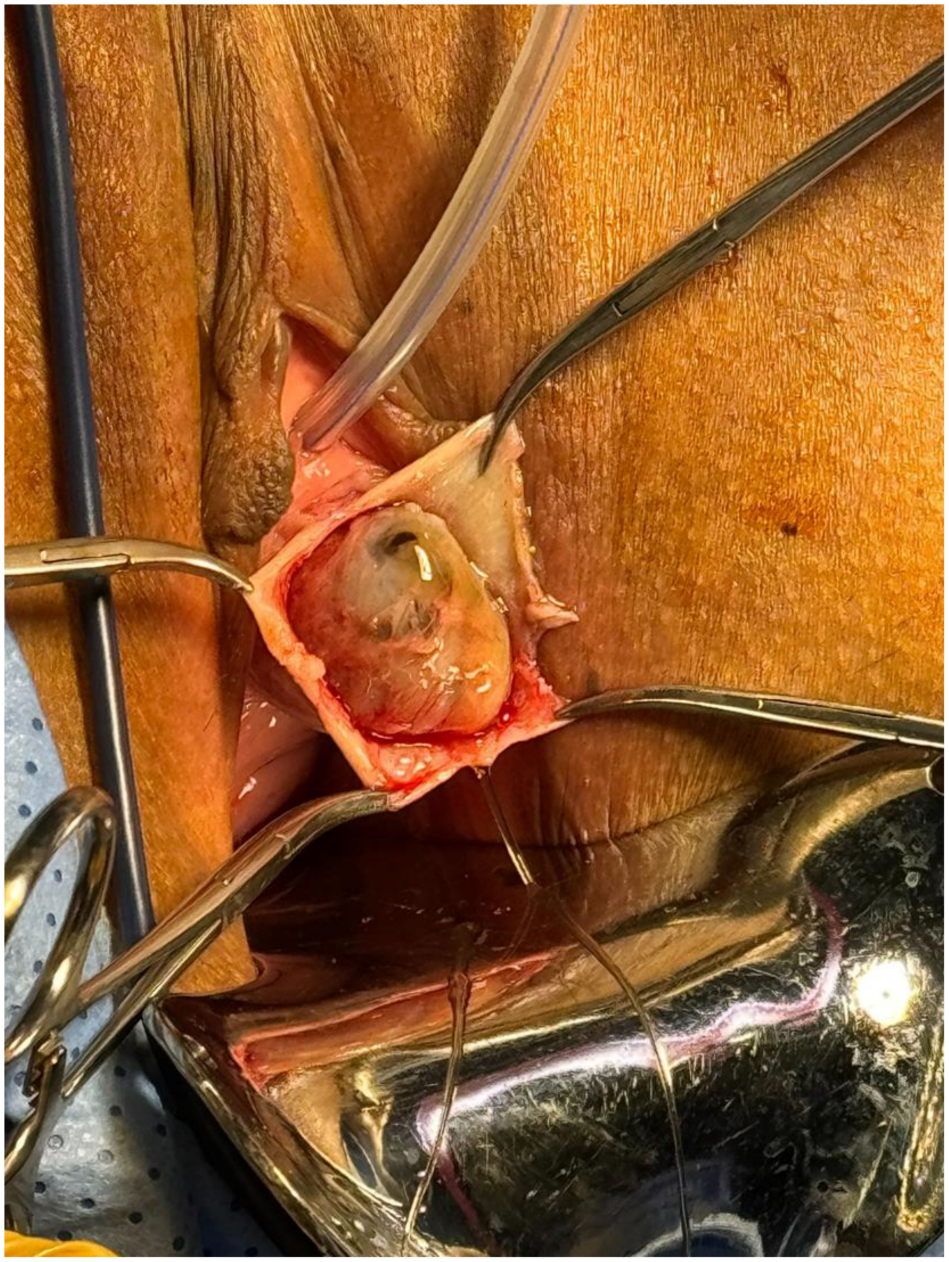
Fully exposed urethral diverticulum with clear delineation of the diverticular wall and its relationship to the urethra prior to complete excision.

In cases where the diverticular cavity was small or difficult to access, normal saline was first injected to outline the sac before instilling the dye. Injection sites were not chosen randomly but were targeted directly over the palpable periurethral mass and its anatomical relation to the urethra, minimizing diffusion distance, which is an important consideration given methylene blue’s slower paracellular diffusion. Confirmed cases underwent transvaginal urethral diverticulectomy under regional or general anesthesia. A midline anterior vaginal wall incision was made over the palpable swelling (**Figure 3**), followed by careful dissection of the vaginal epithelium to expose and mobilize the diverticular wall circumferentially to its neck. The diverticulum was completely excised at the junction with the urethra, if possible, to ensure total removal of the epithelialized tract. The urethral defect was closed in three layers using absorbable sutures. At the end of surgery, a 16-French Foley catheter was inserted and kept *in situ* depending on the location of the diverticulum: distal diverticula: 5 days; mid-urethral or proximal diverticula: 10–14 days. All patients received preoperative antibiotic prophylaxis and continued antibiotics postoperatively for the duration of the Foley catheter.

Excised specimens were sent for histopathological examination, and the presence of urethral mucosa with periurethral glandular or fibrous tissue was considered diagnostic of urethral diverticulum. Patients were followed at 2 and 4 weeks postoperatively and subsequently at 1, 2, and 3 years, either in person or via telephone. Follow-up assessments included evaluation of symptom resolution, continence status, and recurrence.

If recurrence was clinically suspected, patients were re-examined and underwent repeat cystourethroscopy to confirm or exclude residual or recurrent diverticulum.

## Results

A total of 15 female patients were evaluated between 2015 and 2025 for suspected UD. The mean age was 42.4 ± 4.2 years (range, 34–48 years), and the mean diverticular size measured 2.5 ± 0.9 cm (range, 1.3–4.0 cm). The most frequent clinical presentation was recurrent UTI observed in 8 patients (66.7%), followed by a vaginal or periurethral mass in 6 (50%), dyspareunia in 5 (41.7%), and urinary frequency or urgency in 3 (25%). The classic triad of dysuria, dyspareunia, and dribbling occurred in 1 patient (8.3%) (**Table 1**). Diagnosis was achieved by cystoscopy alone in 4 patients (26.7%), cystoscopy with MB testing in 8 (53.4%), and radiologic imaging in 3 patients (20%), including magnetic resonance imaging (MRI) in 2 (13.3%) and CT urogram in 1 (6.6%) (**Table 2**). The anatomic distribution of confirmed diverticula was proximal in 2 cases (16.7%), mid-urethral in 8 (66.6%), and distal in 2 (16.7%) (**Table 2**). All patients underwent transvaginal urethral diverticulectomy with multilayer closure. Postoperative symptom resolution was achieved in 10 patients (83.3%), with no recurrence of urinary infection or voiding dysfunction during early follow-up. Complications occurred in 3 patients (25%), including recurrence in 2 (16.7%) and a vesicovaginal fistula in 1 (8.3%), which resolved with conservative management. No cases of urethral stricture or *de novo* stress urinary incontinence were reported. Follow-up assessments were performed at 2 and 4 weeks and subsequently at 1, 2, and 3 years, showing stable surgical outcomes. In the two recurrent cases, repeat cystourethroscopy confirmed small residual or recurrent diverticula, both of which were successfully re-excised with full recovery. These findings demonstrate the high diagnostic utility and specificity of methylene blue testing as a complementary intraoperative tool when cryptoscopic visualization is inconclusive. Cystourethroscopy alone identified a diverticular ostium in 4 patients (26.7%). In the remaining cases, intraoperative MB dye injection was used to delineate the diverticular communication. Among the 12 patients with histopathologically confirmed UD, the MB test was positive in 11 (91.7%) and negative in 1 (8.3%).

**Table 1:** Characteristics of patients with UD.

Variable	n (%)
Mean age (range)	42.4 ± 4.2 (34–48)
Recurrent urinary tract infection	8 (66.7%)
Vaginal or periurethral mass	6 (50 %)
Dyspareunia	5 (41.7 %)
Frequency/urgency	3 (25 %)
Classic triad (dysuria, dyspareunia, dribbling)	1 (8.3 %)

**Table 2:** Cystourethroscopy and surgical findings.

Parameter	Value
Mean diverticular size, cm (range)	2.5 ± 0.9 (1.3–4.0)
Diagnosis method	
Cystoscopy alone (before using methyl blue)	4 (26.7%)
Cystoscopy + methylene blue test (+ve)	4 (26.7%)
Cystoscopy + methylene blue test (−ve)	4 (26.7%)
MRI	2 (13.3%)
CT urogram	1 (6.6%)
Location by clinical and cystourethroscopy findings	
Proximal	2 (16.7%)
Middle	8 (66.6%)
Distal	2 (16.7%)
Postoperative complications	
Recurrence	2 (16.7%)
Fistula	1 (8.3%)
Mean age (range)	42.4 ± 4.2 (34–48)
Recurrent urinary tract infection	8 (66.7%)
Vaginal or periurethral mass	6 (50%)
Dyspareunia	5 (41.7%)
Frequency/urgency	3 (25%)
Classic triad (dysuria, dyspareunia, dribbling)	1 (8.3%)

In contrast, all three patients with non-diverticular lesions—two with Skene’s gland cysts and one with vaginal mucosa cyst—had a negative MB test (100%). When compared with final histopathology, the MB test showed a diagnostic sensitivity of 91.7%, specificity of 100%, positive predictive value of 100%, negative predictive value of 75%, and overall diagnostic accuracy of 93.3%.

## Discussion

Female UD continues to present diagnostic and management challenges due to its rarity, heterogeneous presentation, and frequent overlap with other lower urinary tract conditions. Consistent with previous literature, the classic triad of dysuria, dyspareunia, and post-void dribbling was uncommon in our cohort (8.3%), reflecting findings from contemporary case series where the triad occurs in <20% of patients (6, 14).

In our series of 12 histologically confirmed cases, methylene blue-assisted cystourethroscopy demonstrated a high specificity (100%) and good sensitivity (91.7%), confirming its value as an intraoperative adjunct when the diverticular ostium is difficult to visualize. These accuracy values are comparable to previously described small cohorts, although large-scale validation remains lacking. In our cohort, some patients had MRI, others underwent CT, while a few had no preoperative imaging at all, reflecting the retrospective nature of the study and the fact that investigations were ordered based on the assessment and preference of the referring physician. Consequently, methylene blue testing was performed routinely intraoperatively to aid localization regardless of prior imaging availability. MRI remains the gold standard for the preoperative evaluation of urethral diverticulum. Contemporary studies consistently demonstrate MRI’s superior ability to define complex configurations such as saddle-shaped, circumferential, multiloculated, or proximally located lesions, which critically influence surgical planning, recurrence risk, and postoperative outcomes (14, 15). In contrast, methylene blue testing cannot offer comparable anatomical resolution, and the lack of consistent MRI correlation in our cohort limits the evaluation of diagnostic concordance, representing an important limitation of this study. Although fluorescein and indocyanine green can provide superior fluorescence-based contrast when advanced imaging systems are available (23), our technique relied on methylene blue because it is simple, inexpensive, widely available, and detectable under standard cystourethroscopy. The methylene blue technique also carries a potential risk of dye extravasation with deep or misplaced injection, which may obscure findings and underscores its operator-dependent nature, although this complication was not encountered in our cases. Given this learning curve, MB testing should be considered an adjunct rather than a replacement for established imaging modalities.

Another important consideration is that although MB testing is proposed as a low-cost technique for resource-limited settings, the procedure in our cohort required sedation or general anesthesia, which may offset the perceived advantages in low-resource environments. Our recurrence rate (16.7%) was slightly higher than in large contemporary series—typically 3%–10% (14, 18), which may reflect the predominance of mid-urethral lesions (66.6%), variability in preoperative imaging, and inclusion of earlier surgical experience. Although contrast-free T2-weighted MRI urography has been shown to aid in detecting postoperative urine leaks in selected high-risk cases, its routine use in our setting is limited by cost, long waiting times, and resource constraints; however, it remains a valuable option for carefully selected complex cases (15, 24, 25).

Overall, while MB-assisted cystourethroscopy offers a simple, real-time, low-cost intraoperative localization tool, particularly when the ostium is not visible endoscopically, it does not replace MRI as the standard diagnostic modality. Its optimal role may be as an adjunct when MRI is unavailable or when intraoperative confirmation of the diverticular neck is required. However, UD, especially complex configurations, should still ideally be managed in tertiary or quaternary centers with subspecialty expertise, and a prospective study directly comparing MRI and methylene blue techniques is needed to more accurately evaluate their diagnostic performance.

## Conclusion

Methylene blue-assisted cystourethroscopy provides a simple and accurate adjunct for intraoperative localization of urethral diverticulum, demonstrating a sensitivity of 91.7% and a specificity of 100%. The technique serves as an adjunct rather than a replacement for MRI, especially in complex cases. Despite its usefulness in resource-limited settings, the study is limited by its small sample size, retrospective design, and inconsistent preoperative imaging. Operator dependence and the requirement for anesthesia may further reduce its practical advantage. Future prospective studies with standardized imaging are needed to validate these findings.

## Data Availability

The original contributions presented in the study are included in the article/supplementary material. Further inquiries can be directed to the corresponding author.
